# High susceptibility, viral dynamics and persistence of South American Zika virus in New World monkey species

**DOI:** 10.1038/s41598-019-50918-2

**Published:** 2019-10-10

**Authors:** Neil Berry, Deborah Ferguson, Claire Ham, Jo Hall, Adrian Jenkins, Elaine Giles, Dhruti Devshi, Sarah Kempster, Nicola Rose, Stuart Dowall, Martin Fritzsche, Thomas Bleazard, Roger Hewson, Neil Almond

**Affiliations:** 1 Division of Infectious Disease Diagnostics, National Institute for Biological Standards and Control, Blanche Lane, South Mimms, Herts EN6 3QG UK; 2 Division of Virology, National Institute for Biological Standards and Control, Blanche Lane, South Mimms, Herts EN6 3QG UK; 3 Virology and Pathogenesis, National Infection Service, PHE-Porton, Manor Farm Rd, Porton Down, Salisbury, SP4 0JG UK; 4 Analytical Sciences Group, National Institute for Biological Standards and Control, Blanche Lane, South Mimms, Herts EN6 3QG UK

**Keywords:** Microbiology, Viral pathogenesis

## Abstract

South American Zika virus (ZIKV) recently emerged as a novel human pathogen, linked with neurological disorders. However, comparative ZIKV infectivity studies in New World primates are lacking. Two members of the *Callitrichidae* family, common marmosets (*Callithrix jacchus*) and red-bellied tamarins (*Saguinus labiatus*), were highly susceptible to sub-cutaneous challenge with the Puerto Rico-origin ZIKV_PRVABC59_ strain. Both exhibited rapid, high, acute viraemia with early neuroinvasion (3 days) in peripheral and central nervous tissue. ZIKV RNA levels in blood and tissues were significantly higher in New World hosts compared to Old World species (*Macaca mulatta*, *Macaca fascicularis*). Tamarins and rhesus macaques exhibited loss of zonal occludens-1 (ZO-1) staining, indicative of a compromised blood-brain barrier 3 days post-ZIKV exposure. Early, widespread dissemination across multiple anatomical sites distant to the inoculation site preceded extensive ZIKV persistence after 100 days in New and Old World lineages, especially lymphoid, neurological and reproductive sites. Prolonged persistence in brain tissue has implications for otherwise resolved human ZIKV infection. High susceptibility of distinct New World species underscores possible establishment of ZIKV sylvatic cycles in primates indigenous to ZIKV endemic regions. Tamarins and marmosets represent viable New World models for ZIKV pathogenesis and therapeutic intervention studies, including vaccines, with contemporary strains.

## Introduction

Zika virus (ZIKV) is a single-stranded RNA virus, a member of the family *Flaviviridae*. Originally isolated from a sentinel rhesus macaque in the Zika Forest, Uganda in the mid-20^th^ century^1^, Zika exists primarily within a sylvatic enzootic transmission cycle between non-human primate (NHP) species and arboreal mosquitoes, predominantly *Aedes sp*., in the African tropics. The ecological balance and relatively benign inter-relationship between virus, mosquito vector and primate species appears to have been disrupted as ZIKV dispersed globally, erupting as a significant human pathogen in the Americas^2^. ZIKV now represents one of a diverse range of globally recognised re-emergent pathogens posing a significant threat to human health.

Identification of ZIKV in Brazil in 2015, its spread through South, Central and parts of North America, correlated with previously unrecognised specific pathology. Notably, severe congenital abnormalities occur in 1–2% infants born to ZIKV-positive mothers infected during pregnancy^3–5^ presenting with microcephaly and impaired brain development^6^, collectively known as congenital Zika syndrome (CZS), but also a range of less severe complications and sequelae which impact on infant and child health. Causality was subsequently confirmed by experimental studies conducted in mice^7^. In adults, ZIKV has been linked to a spectrum of acute neurological conditions, including Guillain-Barré syndrome (GBS) and symptomatic conjunctivitis^8,9^. Non-vector cases of Zika transmission have been described^10^ with ZIKV RNA persisting at high titres in the semen of convalescent healthy blood donors^11^, highlighting the risk of sexual transmission from Zika-positive individuals. The long-term consequences and pathological sequelae of New World ZIKV isolates infecting humans, however, are not fully understood.

Animal models provide valuable insights into disease pathogenesis and support vaccine development. Experimental infection of Old World NHPs with different ZIKV strains has informed our understanding of transmission dynamics and led to improvements in viral diagnostics, therapeutics and vaccines^12–23^. However, given the specific impact of ZIKV in the Americas, information is limited regarding the relative impact and susceptibility of New World monkey species, particularly with coincidental ZIKV isolates circulating in the Americas^24^. Marmosets are reported to be susceptible to experimental infection with an African-lineage ZIKV strain^25^. Retrospective analysis of neotropical NHP carcasses, mainly marmosets and capuchins, in urban tropical locales in Brazil identified Zika genome in over one third^26^, confirming earlier reports of potential for ZIKV to infect species native to South America, including squirrel and owl monkeys^27^. Nevertheless, comparative analyses of differential host susceptibility among New and Old World NHPs with contemporaneous New World ZIKV strains have not been performed. Here, we evaluate virological outcomes in two taxonomically distinct New World species: common marmosets (*Callithrix jacchus*) and red-bellied tamarins (*Sanguinus labiatus*), following sub-cutaneous challenge with the Caribbean-derived ZIKV_PRVABC59_ isolate. Red-bellied tamarins have been previously studied in our laboratory for acute hepacivirus infection, demonstrating susceptibility to flaviviruses^28^. Data from these New World hosts were compared to Old World NHP models represented by two species, Indian rhesus macaque (*Macaca mulatta*, RM) and cynomolgus macaque (*Macaca fascicularis*, CM). Hence, this represents the first report to directly compare viral dynamics and tissue persistence in Old World and New World species infected with the same contemporary ZIKV strain under experimental conditions.

Both New World monkey species were highly susceptible to sub-cutaneous ZIKV_PRVABC59_ inoculation, typified by rapid appearance 1–2 days post-infection (dpi) and high virus load in blood associated with co-incident widespread virus dissemination in secondary lymphoid tissues. In tamarins and RM, although viremia cleared predominantly 15–42 dpi persisting viral RNA (vRNA) signals were detected in multiple tissues and anatomical sites as late as 100 dpi, notably neurological and reproductive tissue. Persisting ZIKV RNA signals demonstrate capacity of this New World isolate to enter the adult central nervous system (CNS) and persist in the absence of clinical symptoms. As early as 3 dpi with ZIKV, evidence of a compromised blood brain barrier (BBB) was identified in both tamarins and RM preceding persisting infection in brain tissue sections up to 100 dpi. Widespread persistence in both New and Old World hosts raises the possibility that a significant proportion of Zika-exposed individuals during the recent outbreak in South America may be harbouring Zika genome in multiple anatomical sites, including neurological tissue, with potential consequences on human health.

High susceptibility of distinct New World monkey species to ZIKV underscores the potential for epidemic-associated ZIKV to establish a sylvatic cycle in indigenous primate species in South America which should be considered when modelling re-emergence of human ZIKV outbreaks in this region. Of most concern are the long-term sequelae of rapid neuroinvasion and persistence of New World ZIKV in adults exhibiting few, if any, clinical signs of infection.

## Results

### Virus stock and study design

To assess susceptibility of different NHP species to a contemporary ZIKV strain recently circulating in the Americas, Old and New World species were compared. Two New World species, common marmosets and red-bellied tamarins and two Old World species, Indian RM and Mauritian cynomolgus macaque (CM) were inoculated via the sub-cutaneous route (back of neck), with a total inoculum dose of 1 × 10^5^ PFU of the Puerto Rico-origin ZIKV_PRVABC59_ strain. Virus was obtained via the CDC, USA, originally isolated from the serum of a patient who had travelled to Puerto Rico in 2015^24^. The PRVABC59 stock used in these studies was established having undergone 3 passages on Vero cells at CDC, one additional passage at the National Collection of Pathogenic Viruses (NCPV, UK) and two subsequent passages on Vero cells at NIBSC. Verification of this ZIKV_PRVABC59_ stock was determined by whole viral genome next generation sequencing (deposited at GenBank MK713748). High sequence identity homology to the reported consensus reference genome KU501215 confirmed viral provenance, with only a single majority nucleotide change identified at position 1964G > T (frequency: ~75%).

**Figure 1:**
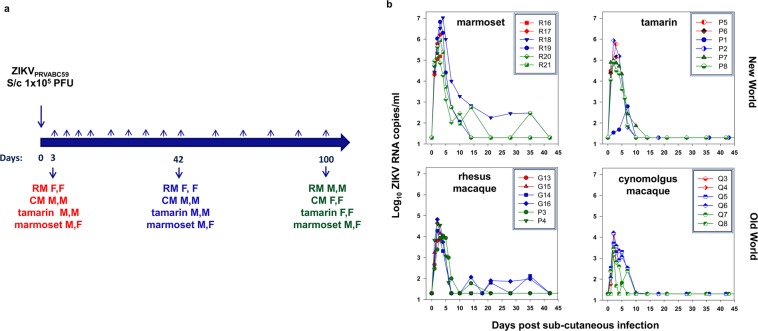
(**a**) Study design. Schematic of study outline with pairs of animals administered virus via the subcutaneous (S/c) route; gender indicated as M (male) or F (female). Rhesus macaques (RM), cynomolgus macaques (CM), red-bellied tamarins (tamarin) or marmosets were challenged and sacrificed after 3 (red), 42 (blue) or 100 days (green). (**b**) Acute viral kinetics in blood post ZIKV PRVABC59 sub-cutaneous inoculation. Viraemia profiles for each time-course experiment with viral RNA levels for each species depicted relating to the day of termination: day 3 (red), day 42 (blue) and day 100 (green), up to and including the day 42 bleed. Reproducibility of sampling for each species shown as a composite representing the different time-courses. Viral RNA levels are expressed as ZIKV RNA copies/ml determined from serum for marmosets and tamarins (New World) and plasma in rhesus and cynomolgus macaques (Old World). All samples assayed beyond 42 days were below the level of detection (50 ZIKV RNA copies/ml).

Animals were bled at regular intervals: daily, weekly and monthly before being euthanased humanely at 3 (acute), 42 (early chronic) and 100 (late chronic) dpi (Fig. 1a). No adverse side effects or deterioration in health condition were identified throughout the study, monitored closely by veterinarian and animal husbandry staff; no rash was observed around the site of inoculation or distally. Haematology remained within the normal range.

### Rapid appearance of ZIKV_PRVABC59_ RNA in blood in New World primates

Single sub-cutaneous administration of 1 × 10^5^ PFU ZIKV_PRVABC59_ (delivered in a total volume of 0.5 ml) resulted in productive infection in all four NHP species. Peripheral vRNA loads (Fig. 1b) revealed high intra-species reproducibility in New and Old World species, peaking 2–4 dpi., though differing in magnitude between species. Marmosets and tamarins displayed the highest levels of ZIKV RNA in blood (RNAemia), comparative day 2 levels of mean log_10_ 4.27 × 10^5^ and 2.92 × 10^5^ ZIKV RNA copies/ml serum respectively, 10–50-fold higher than values obtained for RM and CM (mean log_10_ 3.03 × 10^4^ and 6.88 × 10^3^ ZIKV RNA copies/ml plasma respectively). Comparison at 2 dpi, combining all New World and all Old World data, indicated mean peak viraemia was of at least one order of magnitude higher in New Worlds which was highly statistically significant (*p* = 0.00058, Mann-Whitney U test). Inter-species differences between New and Old World monkeys 2 dpi were also statistically significant, when marmosets were compared individually to RM and CM (both *p* = 0.002, Mann-Whitney test) although tamarins compared to RM and CM was not statistically significant (both *p* = 0.065, Mann-Whitney test). Notably, both New World species displayed a more rapid increase in RNAemia than either Old World species, attaining ~10^4^ ZIKV RNA copies/ml 1 dpi, with one exception, tamarin (P8). Overall, New World monkeys supported high levels of virus replication during the acute phase.

High serum ZIKV RNA in marmosets was followed by more gradual virus clearance from peripheral blood ~28 dpi. In marmoset R18, peak RNAemia occurred 4 dpi., (1.05 × 10^7^ ZIKV RNA copies/ml), declining over the subsequent 3 weeks. In CM and tamarins vRNA signals resolved to undetectable levels 14 dpi. Incidences of recrudescing viraemic episodes after the acute phase were identified in two RM (G14, G16) and one marmoset (R20), with an additional marmoset (R18) failing to clear ZIKV RNA until 42 dpi. Taken together, these data demonstrate both New World primate species to be highly susceptible to ZIKV_PRVABC59_, supporting high peripheral vRNA during the acute phase and all four NHP species studied exhibiting a controlled ZIKV RNAemia to undetectable levels within the first 2 months of infection.

### Anti-Zika serology

All individuals had seroconverted to ZIKV by 42 dpi., although anti-NS-1 binding and neutralisation titres varied considerably between species (Fig. S1), broadly reflecting peripheral vRNA levels. Inter-species comparisons were potentially hampered by secondary antibody recognition rather than true inter-species differences in antibody generation *per se*. As expected, control antigens to chikungunya and dengue viruses were unreactive with all sera by Western blot. All individuals made anti-ZIKV neutralising antibody responses with variable 90% neutralisation titres (NT_90_). Marmosets generated the highest, most consistent NT_90_ responses, CM the lowest, mirroring the acute virus load. Differential serological responses across different assays likely reflect recognition of either conformational or linear epitopes which explains high NT_90_ values with low or absent binding to E protein (Mikrogen RecomLine assay), the latter employing denatured recombinant proteins which detect only linear epitopes of the IgG subclass.

### High ZIKV levels in lymphoid tissues in New World monkeys during acute infection

**Figure 2:**
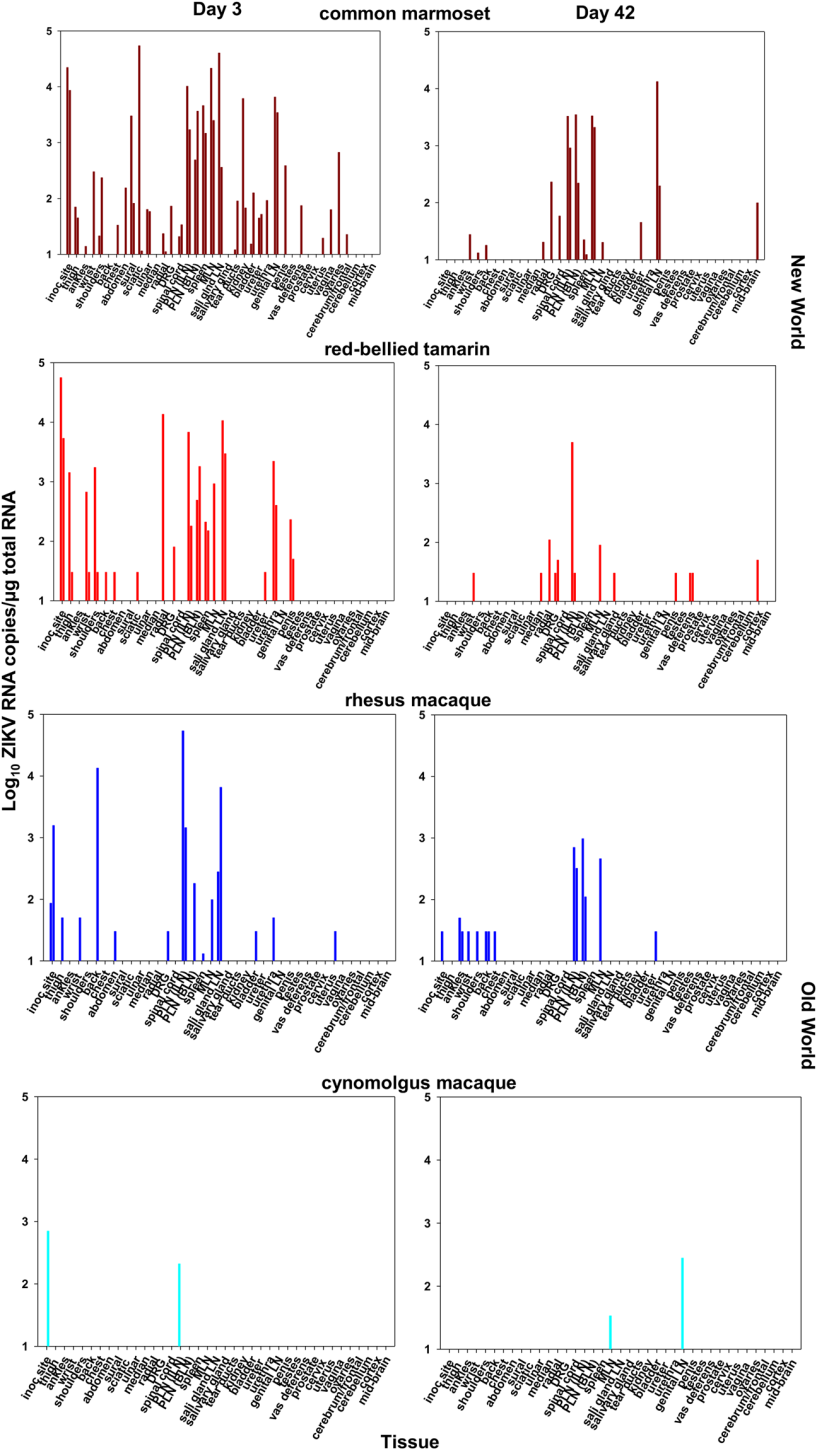
Tissue distribution of ZIKV RNA in New and Old World monkeys determined by qRT-PCR. Tissue viral RNA levels in materials collected *post-mortem* following total tissue RNA extraction, expressed as ZIKV RNA copies per µg total RNA normalised to a GAPDH house-keeping gene. Days 3 and 42 are compared for each species. Tissues are broadly grouped according to anatomical region - skin: (inoculation site, thigh, ankle, wrist, shoulders, back, chest, abdomen); nerve tissue: (sural, sciatic, ulnar, median, radial, dorsal root ganglion (DRG)); Lymph node (LN): peripheral lymph node (PLN), - inguinal lymph node (ILN), basal lymph node (BLN), mesenteric lymph node (MLN), spleen, salivary gland lymph node; secretory sites: salivary gland, tear ducts, kidney, bladder, ureter, urethra; reproductive sites: genital lymph node (LN), penis, testes, vas deferens, prostate, cervix, uterus, vagina, ovaries; brain tissue: cerebrum/frontal lobe, cerebellum, cortex, mid-brain.

**Figure 3:**
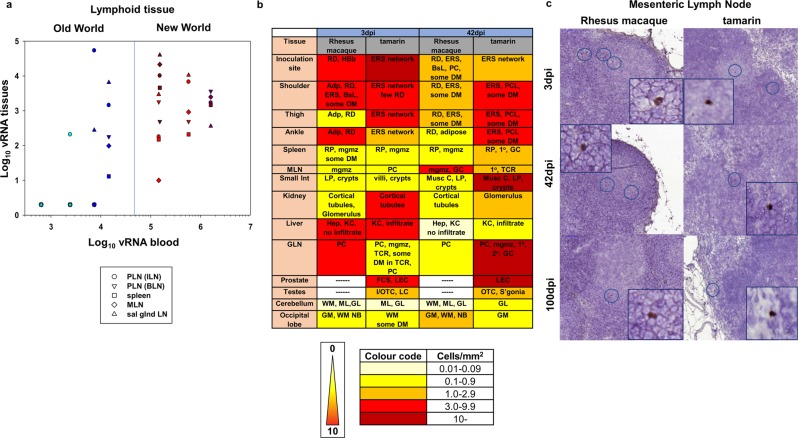
(**a**) Relationship between tissue vRNA and blood vRNA levels in individual Old and New World monkeys in lymphoid tissue 3 days p.i. Symbols representing each tissue sampled are shown in the key. PLN (ILN/BLN); peripheral lymph node (inguinal/basal); MLN, mesenteric lymph node; sal glnd LN (salivary gland lymph node). Colour code: marmosets (dark red), tamarins (red), Indian rhesus macaque (blue) and Mauritian cynomolgus macaque (light blue). (**b**) Comparative RNAscope analysis of ZIKV RNA tissue distribution. The frequency and intensity of staining frequencies are indicated by the heat-map coding as shown. Localised areas of staining summarised in the table are abbreviated as follows: Adp, adipose layer; RD, reticular dermis; HBb, hair bulb base; ERS, external root sheath; BsL, basal layer; ML, molecular layer; GL, granular layer; GM, grey matter; PC, paracortex; mgmz, marginal/mantel zone; 1°, primary follicle; 2°, secondary follicle; GC, germinal centre; Musc C, muscularis circular muscle layer; LP, laminar propria; PCL, prickle cell layer; Hep, hepatocytes; KC, Kupffer cells; DM, dendritic morphology; TCR, T cell regions, FCS, fibrocollagenous stroma; LEC, luminal epithelial cells; I/OTC, inner and outer tubule cells; OTC, outer tubule cells; LC, Leydig cells; S’gonia, spermatagonia; NB, neuronal body. WM, white matter. GLN, genital lymph node, Small Int, small intestine. **(c**) RNAscope localisation of ZIKV RNA in lymphoid tissue (mesenteric lymph node) Representative images of RNAscope detection of ZIKV RNA within FFPE MLN collected *post-mortem* from either Indian rhesus macaques and red-bellied tamarins 3, 42 and 100 dpi. Main image x20, inset x40 magnification. Characteristic brown stained ZIKV RNA positive cells (circled x20 images) identified within follicular germinal centres, marginal zones and paracortical regions of MLN were present 3 dpi remaining detectable 42 and 100 dpi.

Analyses of a wide range of tissues from New and Old World species identified virus sequestration and distribution in lymphoid tissue reflecting the magnitude of the early viraemia (Fig. 2). Both New World species exhibited high cell-associated ZIKV RNA (CA-vRNA) tissue distribution; the highest frequency and magnitude of tissue CA-vRNA sequestration occurred in marmosets 3 dpi including peripheral and mesenteric lymph nodes (PLN/MLN) and spleen; tamarins exhibited comparably wide ZIKV tissue distribution. A direct relationship between ZIKV RNAemia and ZIKV CA-vRNA levels was identified 3 dpi in lymphoid tissue (Fig. 3a), Spearman correlation coefficient r^2^ = 0.51. Comparative tissue viral dynamics across all four species indicated high susceptibility and dissemination of ZIKV_PRVABC59_ among New Worlds, statistically higher compared to Old World in the tissues analysed (p < 0.001, Fig. 3a). These data reflect a strong systemic viral burden during acute infection in New World monkeys, irrespective of the species/genera. In most tissues, ZIKV RNA persisted at lower levels 42 dpi but reflected the tissue distribution 3 dpi (Fig. 2).

### Localisation of ZIKV at 3 dpi correlates with persistence at later times

Complementary RNAscope *in situ* hybridisation (ISH) and qPCR data indicate ZIKV is rapidly established with a widespread and persisting infection in multiple tissues in New World tamarins and marmosets, coincident with primary infection blood dynamics (Fig. 3a–c). Distribution of localised ZIKV RNA infection foci determined by RNAscope established 3–42 dpi in New World tamarins and Old World RM are summarised in Fig. 3b. As with Old World hosts, ZIKV RNA was detected long after resolution of the primary viraemia in the New World hosts indicating widespread persistence across multiple anatomical sites 3–42 dpi. Comparison of the 42 and 100 dpi time-point data indicate this represents a common feature of both tamarins and RM. RNAscope ISH distribution of viral foci 3, 42 and 100 dpi are depicted for mesenteric lymph nodes (MLN, Fig. 3c) and spleen (Fig. S2) representing longitudinal analysis of viral foci, with subtle differences in spatial distribution of ZIKV RNA signals between tamarins and macaques; tamarins exhibited higher virus infiltration around the paracortical regions of the MLN (Fig. 3c). In liver sections, inflammatory infiltrates involving Kupffer cells were identified in tamarins.

### ZIKV detection in secretory tissue

ZIKV RNA shedding was compared in different secretory fluids, including urine, over the 42 day time-course (Fig. S3a). Viruria was more readily detected in tamarins, P1 exhibiting a delayed profile reflecting virus levels in blood. Viral RNA detection in tamarin urine by qRT-PCR was compatible with high staining for ZIKV RNA by RNAscope around cortical tubules of the kidney (Fig. 3b). In macaques, virus was either absent or low in urine, reflecting low RNAscope staining patterns in kidney sections. ZIKV RNA was also detected in various non-lymphatic sites including tear ducts and salivary glands but also salivary gland LN by qRT-PCR and/or RNAscope ISH. Male tamarins (P1, P2) exhibited high levels of staining for ZIKV RNA by RNAscope ISH in tear duct, salivary gland and salivary gland LN 42 dpi (Fig. S3b); ZIKV RNA was not recovered from saliva by qRT-PCR.

### Peripheral ZIKV distribution in skin and nerves

**Figure 4:**
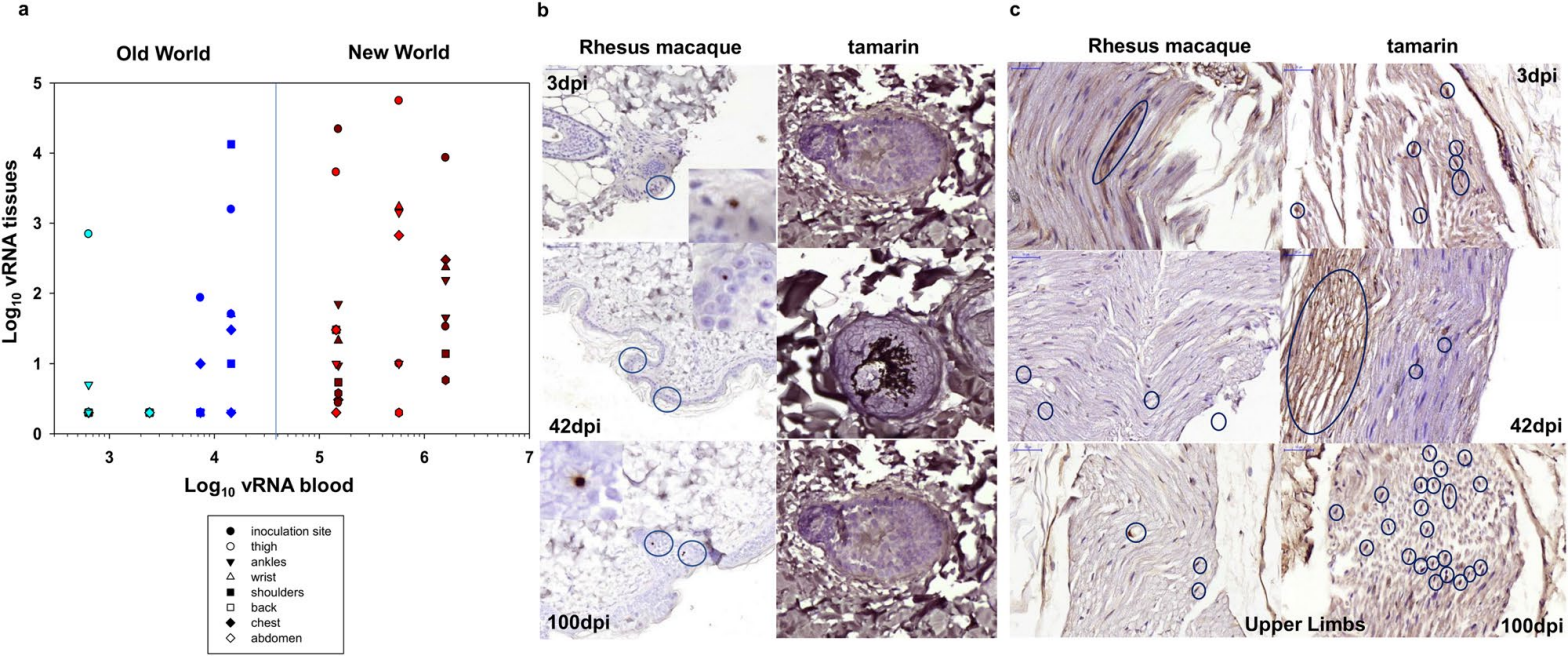
(**a**) Relationship between tissue vRNA and blood vRNA levels in individual monkeys in multiple skin tissues 3 dpi. Symbols representing each tissue sampled are shown in the key. Colour code: marmosets (dark red), tamarins (red), Indian rhesus macaque (blue) and Mauritian cynomolgus macaque (light blue). (**b**) Localisation of ZIKV in skin biopsies. Representative images of RNAscope ISH detection of ZIKV RNA within FFPE skin punch biopsies for rhesus macaque and red-bellied tamarins. Sections were collected from each species from defined sites across the body *post-mortem* 3, 42 and 100 dpi. Images shown are from the inoculation site on the back of the neck (tamarin P1; 42 dpi and tamarin P8, 100 dpi) and ankle (tamarin P5, 3 dpi). Image (x20 magnification), brown stained ZIKV RNA positive cells identified within both epidermal and dermal layers of the skin but primarily in the external root sheath of hair follicles. Numerous ZIKV RNA stained cells with a dendritic cell morphology were present within red-bellied tamarins 100 dpi. (**c)** Immunohistochemical staining for NS-1 protein in peripheral nerves. All images x40 magnification shown for section across areas of upper limb in rhesus macaques (left panels) and tamarins (right panels) at 3, 42 and 100 dpi. Clusters of nerve fibre cells are visible 3 dpi.

As skin is a major target organ for ZIKV, the prevalence and distribution of ZIKV RNA in a diverse range of peripheral anatomical sites was determined (Fig. 4a,b). RNAscope ISH analysis of dermal and epidermal skin layers indicated ZIKV distribution throughout all regions of dermal tissue, particularly external root sheaths of the hair follicles, evident in both tamarins and RM (Fig. 4b). Anti-ZIKV staining, as an antigenic marker of recent infection denoted by the central role the NS-1 protein plays in viral pathogenesis^29^, with an NS-1 antibody indicated viral protein expression around Schwann cells rather than neuronal bodies. Visible in clusters in sections of upper and lower limbs of macaques and tamarins (Fig. 4c, Fig. S4), which correlates broadly with *in vitro* data from neural cell cultures^30^.

High ZIKV CA-vRNA (>5000 ZIKV RNA copies/µg total RNA) was detected in both New World species around the inoculation site with a widely disseminated infection reflected by multiple ZIKV positive skin biopsies 3 dpi. Prevalence and magnitude of ZIKV RNA in multiple skin biopsies was statistically higher overall (p < 0.001, Mann-Whitney U test) in New World compared with Old World species (Figs 2 and 4a), although all species displayed this broad distribution of peripheral virus infection. Comparative ZIKV RNA detection in skin by RNAscope ISH with foci present in RM and tamarins 3, 42 and 100 dpi (Fig. 4b) reflects low but consistent positivity detected in nerve tissue (sural, sciatic, ulnar, medial, radial nerves and dorsal root ganglia) by qPCR (Figs 2 and 4a), although virus was not detected in nerve tissues by RNAscope ISH.

Dendritic cells expressing high levels of DC-SIGN around hair follicles in RM (Fig. S5a) were more pronounced in tamarins (Fig. S5b). Hair follicles are populated by Langerhans cells^31^ which do not express DC-SIGN^32^. The skin samples analysed did not contain large numbers of macrophages in this area, suggestive of the presence of another DC population. Whilst not predominantly detected in hair follicles, CD68+ macrophages were present throughout the dermis with higher levels in tamarins (Fig. S6) in positions co-located with ZIKV-positive cells.

### ZIKV crosses the blood brain barrier 3 dpi and persists in brain tissue in New World monkeys 100 dpi

**Figure 5:**
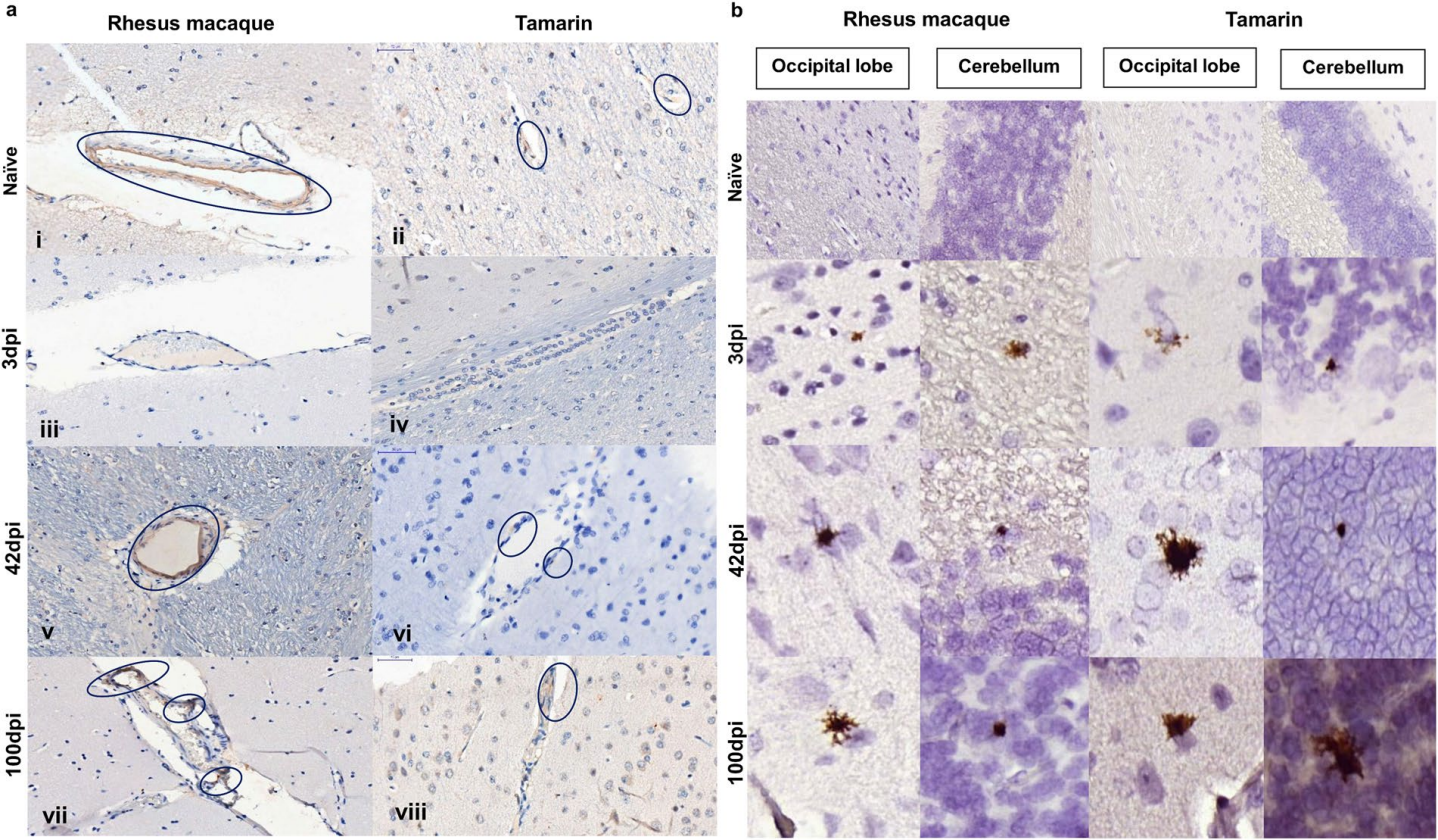
Impact of ZIKV in brain tissue. (**a**) Immunohistochemical staining for the tight junction protein zonula occludens-1 (ZO-1). Characteristic staining within the walls of blood vessels present in white and grey matter of Old and New World primate cerebral cortex. Within the blood vessels of naïve animals, rhesus macaque and tamarin ZO-1 is detected throughout the vessel walls **(i,ii)**. 3 days post infection **(iii,iv)** levels of ZO-1 staining are greatly reduced in both species. By 42 **(v,vi)** and 100 **(vii**,**viii)** days post-infection (dpi) ZO-1 staining is present within blood vessel walls but staining exhibits a fragmented pattern in contrast to continuous staining in naïve animals. (**b**) RNAscope detection of ZIKV RNA in CNS. Representative images within FFPE sections of central nervous system (CNS) of occipital lobe and cerebellum collected *post-mortem* from rhesus macaques or red bellied tamarins 3, 42 or 100 days post-infection (dpi). Comparable distribution of ZIKV RNA positive cells (brown stain, x20 magnification) within the CNS of both Old and New world species. Viral foci detected as early as 3dpi which remained detectable through to 100 dpi. ZIKV positive cells identified within both white and grey matter with a predominantly glial cell morphology associated with neuronal cell bodies.

Using the zonal occludens-1 (ZO-1) marker to assess the ability of ZIKV to compromise the blood brain barrier (BBB), loss of integrity of the endothelial walls of blood vessels within grey/white matter areas was evident 3 dpi in both tamarins and RM (Fig. 5a). This was associated with simultaneous ZIKV RNA detection by RNAscope ISH in multiple regions of these adult brains, including the occipital lobe and cerebellum regions (Fig. 5b). During acute infection ZIKV clearly has the capacity to compromise the BBB integrity leading to establishment of localised infection in different brain regions. ZO-1 staining was largely, though not completely, restored 42–100 dpi to pre-infection levels. Localised ZIKV RNA signals detected in multiple brain regions as early as 3 dpi, which remained detectable 42–100 dpi in both tamarins and RM within white and grey matter regions were associated with multiple cell types. Persistence of virus 100 dpi in CNS tissue of both macaques and tamarins marks a common feature of ZIKV biology in multiple NHP species.

### ZIKV persists in male reproductive tissue in New World tamarins

**Figure 6:**
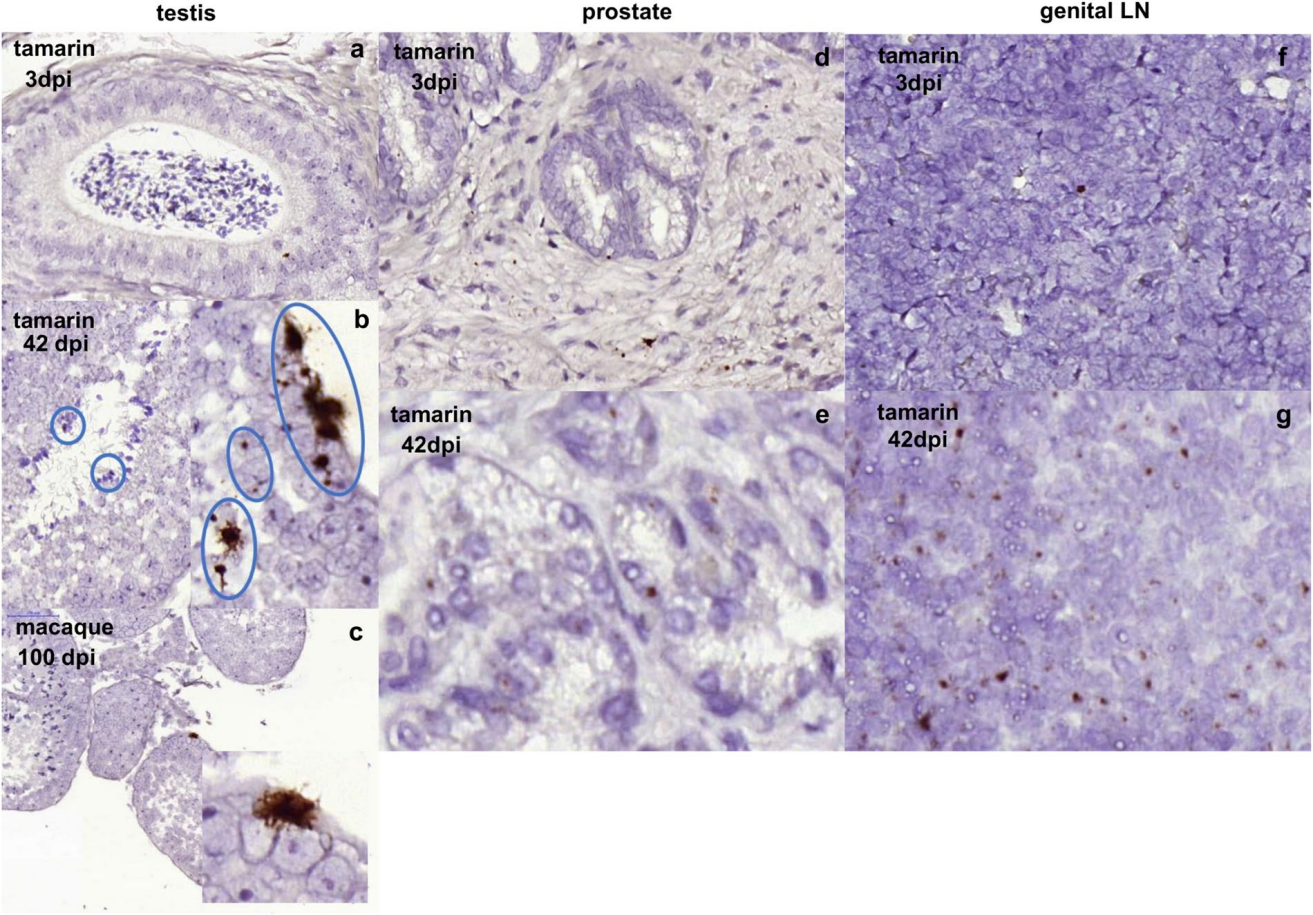
Persistence of ZIKV RNA in male tamarin reproductive tissue. Representative images of RNAscope detection of ZIKV RNA within FFPE tissues from male tamarin reproductive organs collected 3 and 42 days post infection (dpi). Testes sections a–c. (**a)** Main image x20, inset x40 magnification of brown stained ZIKV RNA positive cells identified as discrete foci in seminiferous tubules within sections of testes at 3 dpi (x20 magnification, tamarin P6), with more intense staining out to 42 dpi (panel **b**) with multiple foci covering a more extensive area (tamarin P1). (**c)** Comparative analyses for 100 dpi in the rhesus macaque as a single, large focus of ZIKV RNA expressing cells identified as persistent infection. In both species virus occurs in the outer layers containing primary spermatocytes. Evidence of staining in other parts of male reproductive tissue compared for tamarin P5 (3 dpi) and tamarin P1 (42 dpi) as shown in panels **d,e**) prostate, 3 and 42  dpi, and (**f,g**) genital lymph node (LN), with foci persisting at 3 dpi (tamarin P5) and more widespread at 42 dpi (tamarin P1).

Sexual transmission of ZIKV is associated with high levels of ZIKV RNA in semen up to 6 months after RNAemic clearance, hence it was of interest to determine the persistence of ZIKV in the reproductive tissue of New World hosts. RNAscope ISH analysis identified ZIKV RNA positive cells in male tamarin reproductive tissues of genital LN, prostate and testis (Fig. 6). The majority of the signal within the testis was localised around the periphery of seminiferous tubules in the region of Sertoli cells, indicating a highly localised focus of ZIKV infection in male tamarins as early as 3 dpi, which remained at high levels 42 dpi. Sections of RM testis stained positive 100 dpi confirmed persistence of ZIKV RNA in male macaque reproductive tissue. These data indicate ZIKV RNA persists in the reproductive tissue of New World tamarins, established during the acute infection period.

## Discussion

ZIKV rapidly and unexpectedly entered South and Central America with explosive spread and novel clinical presentations. The role New World NHP hosts may have played during this outbreak is poorly understood, however, susceptibility of different New World NHP species to contemporary ZIKV could have epidemiological consequences^33^. Here, we demonstrate New World species of common marmosets and red-bellied tamarins to be highly susceptible to a Caribbean-derived isolate of ZIKV. Both New World species appeared more susceptible to aggressive productive infection than Old World macaques infected with the same isolate, retaining many of the features of acute human ZIKV infection. Susceptibility of tamarins indicate different New World NHP species indigenous in ZIKV endemic regions capable of supporting a rapidly established and productive ZIKV infection resulting in the establishment of long-term, widespread tissue persistence. ZIKV dynamics studies have suggested a short lifetime of virus-infected cells but which generate sufficient viral particles to sustain productive infection^34^. The magnitude of the rapid, early viraemia in New World hosts translated into a high intensity of sequestered virus in multiple anatomical sites, indicate New World hosts to be highly permissive to ZIKV infection and to therefore represent possible reservoirs of infection in the wild.

The challenge dose of a total of 1 × 10^5^ PFU of virus inoculated via the sub-cutaneous route would seem compatible with recent data suggesting this level of virus may be delivered by *A. aegypti* mosquitos during natural transmission^35^. The dynamics of infection described here therefore likely reflect the infection kinetics of New World species if exposure occurred via mosquito bites in an epidemic scenario involving viraemic individuals. It is interesting that the magnitude of response in New World species exceeded that of the Old World monkeys despite the use of serum rather than plasma to compare viral levels in blood, the former recognised to under-estimate vRNA levels when compared directly to plasma-derived determinations on the same volume of analyte. Comparisons of tissue vRNA loads across the four species, which were normalised according to total RNA content, were not susceptible to such factors. Hence the high levels of virus uptake and dissemination in both New World species from a biologically relevant challenge dose underscore their potentially high susceptibility to infection upon exposure with ZIKV.

Both peripheral and central sites were targeted by Zika in New World hosts. Extensive, early and persisting targeting of skin anatomical sites in tamarins, and by comparison RM, was identified which correlates with susceptibility of both human keratinocytes and Langerhans cells^36^ and NHP dermal fibroblasts^37^ to ZIKV infection *in vitro*. Peripherally located ZIKV-infected cells elicited a strong innate response with early upregulation of DC-SIGN but with no detectable clinical signs of erythema and this response was unable to eliminate all ZIKV RNA positive cells. Clinically, acute ZIKV represents an increased risk for acute peripheral neuropathies, notably GBS in ZIKV endemic regions^38^. Analysis of peripheral nerve fibres detected NS-1 antigen expression 3 dpi, although virus detection in nerve fibres does not define pathogenesis (direct virus damage vs immunopathology) in the absence of clinical signs in any of the NHP models. However, ZIKV appears directly cytopathic in myelinating Schwann cells and peripheral neurons resulting in myelin disruption in IFNAR^−/−^ knockout mice which lack the receptor for type 1 interferon^39^. More detailed analysis of peripheral nerves is required in these immunocompetent models to fully understand their impact on disseminated infection.

Persisting vRNA signals in tissues >60 days further implies onward transmission between cells locally over an extended period. Persistence of viral RNA in neurological tissue, at both peripheral and central sites of the nervous system represents an under-recognised feature of ZIKV biology with potential health implications for ZIKV-exposed individuals during the recent outbreak. Identification of ZIKV RNA in adult brain tissue 3 dpi was particularly striking, given ZIKV_PRVABC59_ is not specifically adapted to replicate in neurological tissue, although embryonic mouse CNS studies indicate potentially all ZIKV strains are likely capable of reproducing the neurovirulent phenotype if the CNS is penetrated. Although not selected as a neurotropic virus, ZIKV_PRVABC59_ infection resulted in extensive loss of ZO-1 staining 3 dpi and widespread virus detection in the adult brain. *In vitro* studies and those conducted in neonatal mice indicate persistence of viral nucleic acid in brain tissue is unlikely to be due to repeated waves of neuroinvasion^40,41^. Demonstration here of prolonged persisting RNA signals 60 days after BBB repair would concur. The long-term impact of ZIKV infection on brain tissue needs careful consideration as acute injuries are known to initiate long term neuroinflammatory and ultimately accelerated neurodegenerative conditions.

The capacity of ZIKV CA-vRNA to persist in critical organs highlights the long-term health implications of the ZIKV outbreak in the Americas. Infection of a large immunologically naïve population almost simultaneously may highlight chronic health problems not apparent in endemic countries. Of concern in adult populations is persistence of an otherwise acutely resolving pathogen in neurological and reproductive tissue. ZIKV persistence within male reproductive sites has resulted in sexual transmission many months after resolution of viraemia^42^. Sertoli cells, important in spermatogenesis within the male testis represent a key cell-type supporting ZIKV replication and viral reservoir^43^, manifesting significant host transcriptional dysregulation during acute infection. ZIKV RNAscope ISH signals in male tamarin testis 3–42 dpi and RM 100 dpi signify replicative virus during the early stages of ZIKV entry and prolonged persistence. Clearly, persisting RNA signals do not demonstrate viable virus. However, reservoir-derived virus may remain infectious many months after ZIKV diagnosis, identified as a risk of sexual transmission^44^. Transplant-related transmission from solid organ donors obtained from non-viraemic donors to immunocompromised hosts^45^ further indicate the potential for on-going ZIKV replication in the liver and kidney. Virus infectivity studies determining the ability to recover viable virus from tissues considered to be in an immunoprivileged position would clarify the biological significance of low level, persisting RNA-positive signals. However, the techniques used to derive these data suggest the virus is transcriptionally active at some level yet able to evade local immune surveillance systems. Understanding the mechanism of persistence in such locations would be informative.

The ability of this New World ZIKV isolate to infect indigenous species of neotropical primates highlight potential for Zika to establish infection reservoirs in multiple NHP species^26,27,37,46^. Tamarins (*Saguinus spp*) represent the most diverse and widespread distribution of the Callitrichine genus with red-bellied tamarins indigenous to NW Bolivia, SE Peru and Brazilian Amazonia. Characterisation of ZIKV dynamics in two distinct genera underscores the high susceptibility of neotropical primates to the virus and if transmission can be formally demonstrated will likely impact on the future ecology and natural history of ZIKV infections in the region. Although the prevalence and incidence of human ZIKV in the Americas has declined rapidly since 2015^47^, development of NHP reservoirs may provide a source for resurgent outbreaks.

Establishment of mosquito transmission models using New World NHPs, as developed for RM^15^, would be one way to more formally determine the possibility of establishing sylvatic ZIKV cycles in the Americas. Although difficult to perform these studies would enable evaluation of vector competence of different New World mosquito species, most relevantly those able to impact on and maintain sylvatic cycles (*Sabethes spp*.; *Haemogogus spp*.), including those capable of bridging sylvatic and urban cycles (*e.g*., *Aedes albopictus*)^33^, given the prevalence of ZIKV genome in neotropical NHPs in these geographical locales^26^. Our study clearly demonstrate that provided infection is established in these New World hosts, they appear fully capable of supporting high levels of acute infection which persists for prolonged periods. We selected a viral dose to challenge the New World species to maintain consistency with other NHP studies and to take into account theoretical considerations of mosquito inoculum delivery. Lower inoculum doses to mimic the spectrum of infectious doses potentially deliverable by a mosquito^35^ which might influence acute infection dynamics and persistence in these New World hosts warrants further study.

The potential for ZIKV to persist asymptomatically in New World species requires consideration in future modelling of ZIKV epidemiology, accounting for data derived from different models^48^. Clinical manifestations of asymptomatic ZIKV infection are further highlighted in recent studies of foetal-ZIKV infection^17^. Long-term sequelae and involvement of asymptomatic ZIKV infection in the adult brain and male reproductive sites following ZIKV exposure has implications for neurological and reproductive site pathology, as most adults exposed to ZIKV remain asymptomatic. With no vaccine currently available, understanding parameters governing long-lived protective immunity will facilitate effective therapy development. In a rapidly changing world where environmental factors, animal welfare and human health are inextricably linked, finding common one-health solutions to these issues are crucial.

## Methods

### Ethical statement

All animal procedures were performed in strict accordance with UK Home Office guidelines, under licence 70/8953 granted by the Secretary of State for the Home Office which approved the work described. Purpose-bred, weaned, red-bellied tamarins were group housed in same-sex groups for the duration of the study, with daily feeding and access to water *ad libitum*. Regular modifications to the housing area and environmental enrichment of all study NHPs were made by husbandry staff. Environmental temperature was appropriate for tamarins and rooms subject to 12 hr day/night cycles of lighting. Animals were acclimatised to their environment and deemed healthy by the named veterinary surgeon prior to inclusion on the study. All surgical procedures were performed under anaesthesia with recovery.

### Virus and study design

PRVABC59 ZIKV strain (GenBank: KU501215) was used in all studies, obtained from National Collection of Pathogenic Viruses, PHE, UK. Virus stocks were prepared by propagation on Vero cells and back titrations performed to verify virus titres from an original stock titre of 6.62 × 10^6^ plaque forming units (PFU). The catalogue number for the stock from NCPV is 1604131 v, Zika virus, strain PRVABC59 (lot#1426). No onward culture of virus or adaptation on macaque or tamarin cells prior to inoculation was performed. Pairs of male or female rhesus macaques, Mauritian-derived cynomolgus macaques, red-bellied tamarins or marmosets were inoculated subcutaneously with 1 × 10^5^ PFU ZIKV_PRVABC59_ administered in a total volume of 0.5 mL. To assess species-specific distribution of ZIKV, two animals from each species were sacrificed 3, 42 or 100  dpi. At termination, an extensive *post-mortem* tissue list was prepared and samples processed for qRT-PCR and RNAscope ISH analyses.

### Sample collection

Sequential blood samples at the time points shown in Fig. 1a were collected from macaques into EDTA (vacuette tubes, Greiner bio-one), centrifuged to obtain plasma, aliquoted and frozen immediately at −80 °C. Serum was isolated by centrifugation from blood collected without anti-coagulant allowed to clot for 4–16 hrs. Early morning urine was collected passively from cage pans at the start of each day minimising biodegradation with as short a time period as possible between urination and sample collection. Samples were centrifuged to remove particulate contaminants, supernatant aliquoted and frozen immediately. Saliva was collected by aspiration with a syringe at the lip, centrifuged to remove food particulates and diluted 1:1 with RNA stabilization reagent (RNA*later*, Qiagen). Faeces were preserved 1:1 (volume) with RNA stabilization reagent for 4–6 hours. Tissues were treated in an analogous manner as previously described^49^; all samples were stored at −80 °C until processed.

### Sample processing

Viral RNA was extracted from 140 μL plasma or serum using QIAamp viral RNA extraction mini-kits (Qiagen Ltd) and eluted with 60 μL RNase-free water. Urine and saliva were concentrated (Amicon Ultra-2, Millipore) before RNA extraction. Samples were eluted in 30 μL RNase-free water/mL of urine, stored at −80 °C until assayed. 40–50 mg tissue was disrupted and homogenised using a bead mill Tissue Lyser (Qiagen Ltd). Total RNA was extracted using an RNA Plus Universal mini kit (Qiagen Ltd). Tissue RNA quantified by nanodrop spectrophotometry was stored at −80 °C.

### Viral sequencing

Full length deep sequence of the PRVABC59 isolate was performed using primers to derive overlapping amplicons spanning the entire genome. Amplicons verified by agarose gel electrophoresis were quantified using Qubit dsDNA HS fluorimetry (ThermoFisher Scientific Ltd). Bar-coded libraries of amplicons were made using Nextera XT library preparation kit, equimolar amounts of each sample sequenced in 250 bp paired-end MiSeq V500 sequencing reactions (both Illumina).

### ZIKV detection in blood and body fluids by qRT-PCR

ZIKV RNA was quantified by qRT-PCR in blood and selected urine, saliva and faecal samples expressed as ZIKV RNA copies/ml analyte. One-step qRT-PCR was based on previously published ZIKV-specific primer sequences^50^ using forward (ZIKV 1086) and reverse (ZIKV 1162c) PAGE-purified primers (Eurofins). Probe sequence (ZIKV 1107-FAM) was as reported^50^ but quenched with 3’ Iowa Black to further reduce background fluorescence using a second, internal ZEN quencher (Integrated DNA Technologies Ltd). ZIKV RNA was assayed in triplicate using Ultrasense one-step qRT-PCR system (ThermoFisher Scientific) in 50 μL reactions containing 5 μL purified RNA from either plasma, serum, urine or saliva with primers and probe at 300 and 50 nM respectively, adopting the cycling conditions previously described^12^. Copy number values were determined against the ZIKV_PRVABC59_ reference virus grown on Vero cells (WHO) (ECACC:88020401) diluted in negative macaque plasma and using Poisson statistics on multiple replicate reactions to determine the assay lower limit (50 ZIKV RNA copies/ml). Standard curves from multiple runs (*n* = 30) gave a slope of −3.3481 (efficiency 98.9%), CV% of 2.01 and *r*^2^ of 0.996. Additional validatory data was obtained using a co-processed ZIKV RNA run control (NIBSC code 16/124), (mean Ct 29.7, CV% 3.13). Intracellular or cell-associated ZIKV RNA was assessed by co-amplification with GAPDH housekeeping gene sequences as previously described^49^.

### RNAscope *In Situ* Hybridisation (ISH) detection of ZIKV RNA within tissues

Samples covering a range of tissue compartments including main organs, lymphoid tissues, reproductive system, peripheral and central nervous system, including the brain and skin were collected at *post-mortem*, fixed in 10% formal saline (Sigma) and embedded in paraffin wax (VWR) using previously reported procedures^51^. Four micron thick sections were mounted on poly-L lysine coated slides (VWR) and prior to treatment de-waxed in xylene and re-hydrated via graded ethanol:water solutions. *In situ* ZIKV RNA detection was performed using the RNAscope 2.5 HD manual DAB detection system (Advanced Cell Diagnostics, 322300) and a combination of two ZIKV-specific probes (463781 and 464531) in accordance with manufacturer’s instructions. Negative (DaPB 310043) and positive (Hs-PPIB 313901) control probes were used to assess technique efficiency. Equivalent tissue sections from infection naïve RM and tamarins were stained with ZIKV-specific, positive or negative control probes to determine NHP tissue cross-reactivities. Sections were manually counter stained with haematoxylin. RNAscope positive cells were quantified by counting all positive cells within each tissue section and conversion to mean number of positive cells/mm^2^. Grading definitions were generated using 10 random fields of view (x10 lens and x10 eye-piece magnification; equivalent to an area of 2.2 mm^2^).

### Statistics

Comparisons of viral load measurements during acute infection were made using Mann-Whitney U non-parametric methods and Spearman correlation co-efficient statistics. Data were plotted graphically using the SigmaPlot 12 analysis package and GraphPad Prism.

### Serology

Anti-ZIKV serology was assessed by three methods at day 42; binding IgG levels, neutralising antibody titres and line immunoassays for tropical fever IgGs. Total anti-ZIKV IgG levels were determined by ELISA (D-23560, Euroimmun Ltd) according to the manufacturers’ instructions. Briefly, serum was diluted 1/100 with diluent before incubation on strips coated with NS-1 antigen for 1 hour at 37 °C. Bound antibody was detected by conjugated secondary antibody and substrate addition. Relative units (RU) were calculated from 450 nm absorbance with subtraction of Abs 630 nm using kit calibrants 1–3 to generate a standard curve. Sera were tested with the RecomLine Tropical Fever IgG immunoassay (Mikrogen Diagnostik Ltd) for NS-1 and E antigens. Neutralisation titres were determined on Vero cells seeded at 3.2 × 10^4^/well of 96 well trays, settled overnight. Heat inactivated serum (56 °C, 1 hour) was serially diluted in MEM without serum and incubated with PRVABC59 for 1 hour at 37 °C. Virus and antibody was added to cells in triplicate at an MOI of 0.005, incubated for 5 days and CPE scored; NT_90_ values were calculated using the Reed and Muench method.

## Supplementary information

Supplementary Information

## References

[1] Dick GW, Kitchen SF, Haddow AJ (1952). Zika virus I. Isolations and serological specificity. Trans R Soc Trop Med Hyg.

[2] Gatherer D, Kohl A (2016). Zika virus: a previously slow pandemic spreads rapidly through the Americas. J Gen Virol.

[3] Driggers RW (2016). Zika virus infection with prolonged maternal viraemia and fetal brain abnormalities. N Engl J Med.

[4] Brasil P (2016). Zika virus outbreak in Rio de Janeiro, Brazil: Clinical characterisation, epidemiological and virological aspects. PLoS Negl Trop Dis.

[5] Clavet G (2016). Detection and sequencing of Zika virus from amniotic fluid of foetuses with microcephaly in Brazil: a case study. Lancet Infect Dis.

[6] Mlakar J (2016). Zika virus associated with microcephaly. N Engl J Med.

[7] Cugola FR (2016). The Brazilian Zika virus strain causes birth defects in experimental models. Nature.

[8] Brasil P (2016). Guillain-Barré syndrome associated with Zika virus infection. Lancet.

[9] Cao-Lormeau VM (2016). Guillain-Barre syndrome outbreak associated with Zika virus infection in French Polynesia: a case-control study. Lancet.

[10] Foy BD (2011). Probable non-vector-borne transmission of Zika virus, Colorado, USA. Emerg Infect Dis.

[11] Musso D (2017). Detection of Zika virus RNA in semen of asymptomatic blood donors. Clin Microbiol Infect.

[12] Dudley DM (2016). A rhesus macaque model of Asian-lineage Zika virus infection. Nat Commun.

[13] Osuna CE (2016). Zika viral dynamics and shedding in rhesus and cynomolgus macaques. Nat Med.

[14] Coffey LL (2017). Zika Virus Tissue and Blood Compartmentalization in Acute Infection of Rhesus Macaques. PLoS One.

[15] Dudley DM (2017). Infection via mosquito bite alters Zika virus tissue tropism and replication kinetics in rhesus macaques. Nat Commun.

[16] Aid M (2017). Zika Virus Persistence in the Central Nervous System and Lymph Nodes of Rhesus Monkeys. Cell.

[17] Dudley DM (2018). Miscarriage and stillbirth following maternal Zika virus infection in nonhuman primates. Nat Med.

[18] Hirsch AJ (2017). Zika Virus infection of rhesus macaques leads to viral persistence in multiple tissues. PLoS Pathog.

[19] Carroll T (2017). Zika virus preferentially replicates in the female reproductive tract after vaginal inoculation of rhesus macaques. PLoS Pathog.

[20] Newman CM (2017). Oropharyngeal mucosal transmission of Zika virus in rhesus macaques. Nat Commun.

[21] Nguyen SM (2017). Highly efficient maternal-fetal Zika virus transmission in pregnant rhesus macaques. PLoS Pathog.

[22] Abbink P (2017). Durability and correlates of vaccine protection against Zika virus in rhesus monkeys. Sci Transl Med.

[23] Haddow AD (2017). High Infection Rates for Adult Macaques after Intravaginal or Intrarectal Inoculation with Zika Virus. Emerg Infect Dis.

[24] Lanciotti R (2016). Phylogeny of Zika Virus in Western Hemisphere, 2015. Emerg Infect Dis.

[25] Chiu CY (2017). Experimental Zika Virus Inoculation in a New World Monkey Model Reproduces Key Features of the Human Infection. Sci Rep.

[26] Terzian AC (2018). Evidence of natural Zika virus infection in neotropical non-human primates in Brazil. Sci Reps.

[27] Vanchiere JA (2018). Experimental Zika virus Infection of Neotropical Primates. Am J Trop Med Hyg.

[28] Hood S (2014). Immune cell changes in the periphery and liver of GBV-B-infected and convalescent red-bellied tamarins (*Saguinus labiatus)*. Virus Res.

[29] Xia H (2018). An evolutionary NS1 mutation enhances Zika virus evasion of host interferon induction. Nat Commun.

[30] Cumberworth SL (2017). Zika virus tropism and interactions in myelinating neural cell cultures: CNS cells and myelin are preferentially affected. Acta Neuropathol Commun.

[31] Nagao K (2012). Stress-induced production of chemokines by hair follicles regulates the trafficking of dendritic cells in skin. Nat Immunol.

[32] Soilleux EJ, Coleman N (2001). Langerhans cells and the cells of Langerhans cell histiocytosis do not express DC-SIGN. Blood.

[33] Althouse BM (2016). Potential for Zika Virus to Establish a Sylvatic Transmission Cycle in the Americas. PLoS Negl Trop Dis.

[34] Best K (2017). Zika plasma viral dynamics in nonhuman primates provides insights into early infection strategies. Proc Natl Acad Sci.

[35] Azar SR, Diaz-Gonzalez EE, Danis-Lonzano R, Fernandez-Salas I, Weaver SC (2019). Naturally infected Aedes aegypti collected during a Zika virus outbreak have viral titres consistent with transmission. Emerg Microbes Infect.

[36] Hamel R (2015). Biology of Zika Virus Infection in Human Skin Cells. J Virol.

[37] Ding Q (2018). Species-specific disruption of STING-dependent antiviral cellular defences by the Zika virus NS2B3 protease. Proc Natl Acad Sci.

[38] Dirlikov E (2018). Clinical Features of Guillain-Barré Syndrome With vs Without Zika Virus Infection, Puerto Rico, 2016. JAMA Neurol.

[39] Volpi VG (2018). Zika Virus Replication in Dorsal Root Ganglia Explants from Interferon Receptor1 Knockout Mice Causes Myelin Degeneration. Sci Rep.

[40] Papa MP (2017). Zika Virus Infects, Activates and Crosses Brain Microvascular Endothelial Cells without Barrier Disruption. Front Microbiol.

[41] Manangeeswaran M, Ireland DD, Verthelyi D (2016). Zika (PRVABC59) Infection Is Associated with T cell Infiltration and Neurodegeneration in CNS of Immunocompetent Neonatal C57Bl/6 Mice. PLoS Pathog.

[42] Joguet G (2017). Effect of acute Zika virus infection on sperm and virus clearance in body fluids: a prospective observational study. Lancet Infect Dis.

[43] Kumar A (2018). Human Sertoli cells support high levels of Zika virus replication and persistence. Sci Rep.

[44] Nicastri E (2016). Persistent detection of Zika virus RNA in semen for six months after symptom onset in a traveller returning from Haiti to Italy. Euro Surveill.

[45] Aguilar C, Husain S, Lortholary O (2018). Recent advances in understanding and managing infectious diseases in solid organ transplant recipients. F1000 Res.

[46] Favoretto SR (2019). Zika virus in Peridomestic Neotropical Primates, Northeast Brazil. Ecohealth.

[47] Ferguson NM (2016). Epidemiology. Countering the Zika epidemic in Latin America. Science.

[48] Osuna CE, Whitney JB (2017). Nonhuman Primate Models of Zika Virus Infection, Immunity and Therapeutic Development. J Infect Dis.

[49] Ferguson D (2014). Early biodistribution and persistence of a protective live attenuated SIV vaccine elicits localised innate responses in multiple lymphoid tissues. PLoS One.

[50] Lanciotti RS (2008). Genetic and serologic properties of Zika virus associated with an epidemic, Yap State, Micronesia, 2007. Emerg Infect Dis.

[51] Ferguson D, Clarke S, Berry N, Almond N (2016). Attenuated SIV causes persisting neuroinflammation in the absence of a chronic viral load and neurotoxic antiretroviral therapy. AIDS.

