# Supplementary material for: High susceptibility, viral dynamics and persistence of South American Zika virus in New World monkey species
Source: Sci Rep. 2019 Oct 10;9:14495. doi: 10.1038/s41598-019-50918-2 (PMC6787206; doi:10.1038/s41598-019-50918-2)
Supplement: Supplementary file 1 — Supplementary Information [file 41598_2019_50918_MOESM1_ESM.pdf]

Supplementary Information

High susceptibility, viral dynamics and persistence of South American Zika virus in New World monkey species

Neil Berry[1*], Deborah Ferguson[1*], Claire Ham[1], Jo Hall[1], Adrian Jenkins[1], Elaine Giles[1], Dhruti Devshi[1], Sarah Kempster[1], Nicola Rose[2], Stuart Dowall[3], Martin Fritzsche[4], Thomas Bleazard[4], Roger Hewson[3], Neil Almond[1].

Division of Infectious Disease Diagnostics[1], Division of Virology[2], Analytical Sciences Group[4], National Institute for Biological Standards and Control, Blanche Lane, South Mimms, Herts. UK. EN6 3QG.

Virology and Pathogenesis, National Infection Service, PHE-Porton[3], Manor Farm Rd, Porton Down, Salisbury SP4 0JG. UK.

*These authors contributed equally

Correspondence: neil.berry@nibsc.org

Figure S1a. Zika anti-NS-1 IgG titres. Zika virus-specific IgG levels expressed as relative units per millilitre (RU/mL) serum for Indian rhesus macaques (Rh), tamarins (Tam), cynomolgus macaques (Cyno) and marmosets (Marm) for the 42 and 100 days post-infection (dpi) time-course showing bleeding frequencies from the start of each study (day 0). All animals seroconverted with relative reactivity indicated, expressed as Relative Units (RU/mL). M, male; F, female.

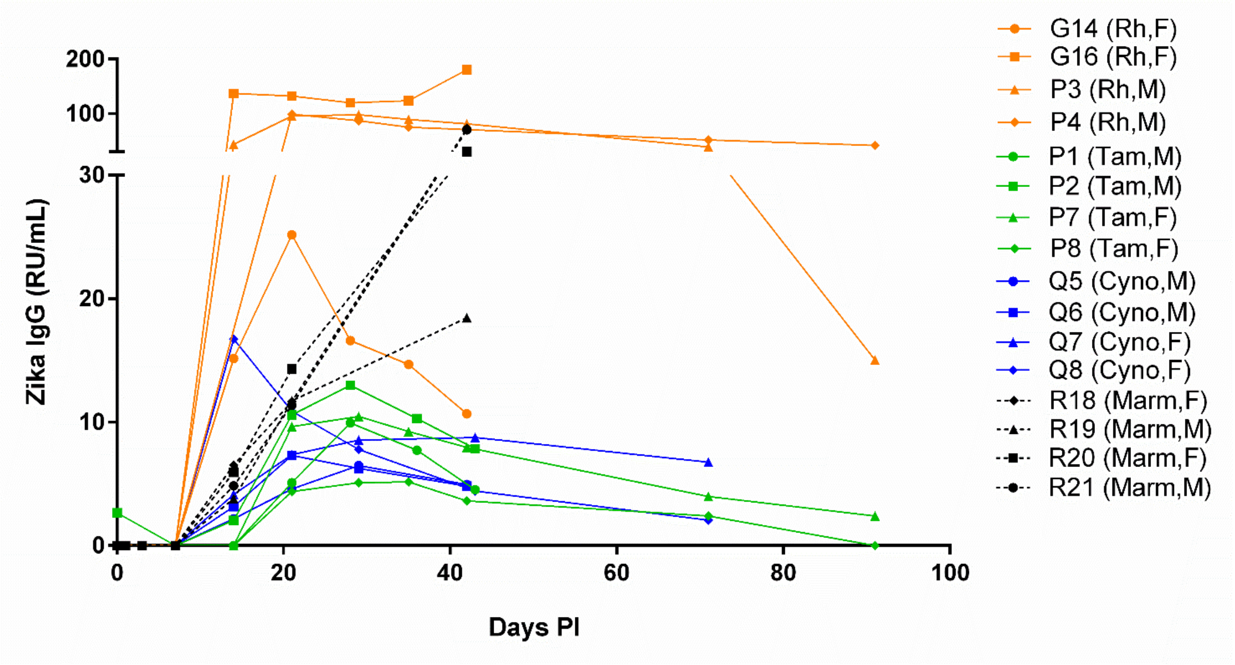

Figure S1b. Comparison of three independent assays of anti-Zika/Flavivirus responses. Rhesus macaques (Rh), tamarins (Tam), cynomolgus macaques (Cyno), marmosets (Marm); convalescent sera taken males (M), females (F) from day 42 (G14, G16, P1, P2, Q5, Q6, R18, R19) or day 100 animals time-course animals (P3, P4, P7, P8, Q7, Q8, R20, R21), all assayed at the day 42 time-point. Details of EuroImmune ZIKV IgG, RecomLine and 90% neutralisation titres (NT_{90}) assays are given in Methods. +, ++ and +++ indicate relative staining intensities in the RecomLine assays for anti-NS-1 and anti-E reactivity.

ID	Euroimmun ZIKV IgG (RU/mL)	RecomLine Tropical ZIKV NS-1	ZIKV E	NT_{90}
**G14 (Rh,F)**	10.67	+	-	139
**G16 (Rh,F)**	180.77	+++	++	2740
**P3 (Rh,M)**	81.52	+++	-	732
**P4 (Rh,M)**	70.75	+++	-	884
**P1 (Tam,M)**	4.54	+	-	68.1
**P2 (Tam,M)**	7.85	+	-	544
**P7 (Tam,F)**	7.96	++	-	432
**P8 (Tam,F)**	3.64	++	+	266
**Q5 (Cyno,M)**	4.96	+	-	54
**Q6 (Cyno,M)**	4.82	+	-	90
**Q7 (Cyno,F)**	8.76	++	-	221
**Q8 (Cyno,F)**	4.42	-	-	40
**R18 (Marm, F)**	73.15	+++	+	1690
**R19 (Marm, M)**	18.45	+++	+	915
**R20 (Marm, F)**	30.16	+++	-	1730
**R21 (Marm, M)**	70.55	+++	-	2745

Figure S2. RNAscope localisation of ZIKV RNA in lymphoid tissue (spleen) Representative images of RNAscope detection of ZIKV RNA within formalin-fixed paraffin embedded (FFPE) sections of spleen collected *post-mortem* from Indian rhesus macaques and red-bellied tamarins analysed 3, 42 and 100dpi. Main image x20 magnification, inset x40.

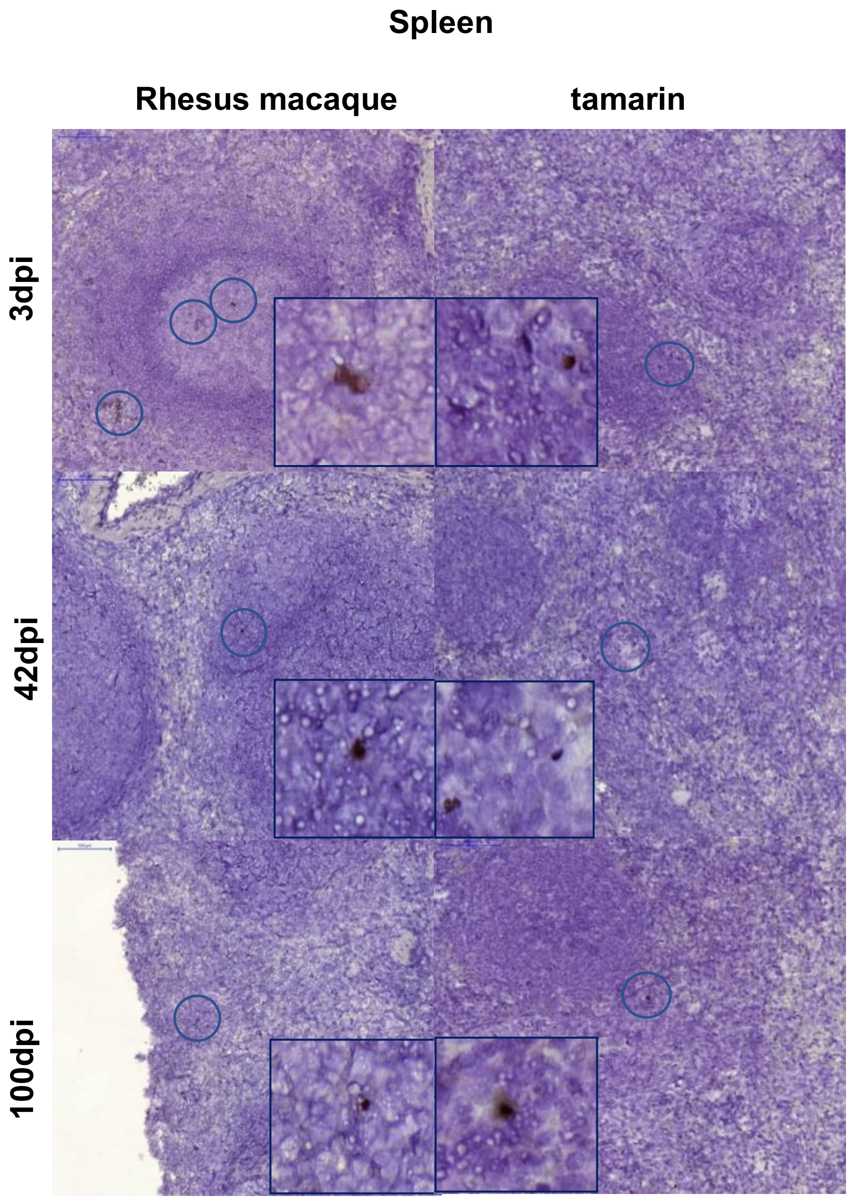

Figure S3a. Viruria levels up to 42 days p.i. in rhesus macaques, tamarins and cynomolgus macaques. Virus load was expressed as ZIKV RNA copies/mL urine extracted. Rhesus macaques (G14, G16); tamarins (P1, P2); cynomolgus macaques Q5, Q6.

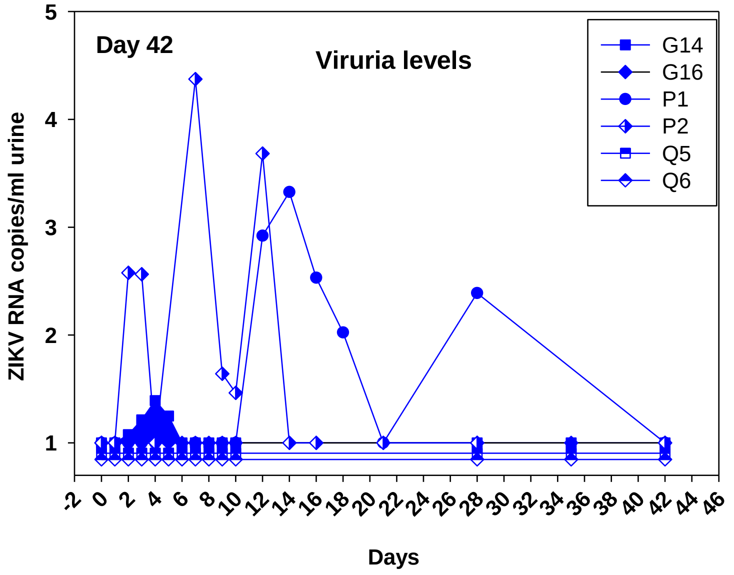

Figure S3b. RNAscope detection of ZIKV RNA in tear ducts and salivary tissue. Comparative ZIKV localisation by RNAscope at day 42 p.i. in male (M) Indian rhesus macaques and female (F) red-bellied tamarins in **i)** tear duct, **ii)** salivary gland and **iii)** salivary gland lymph node (LN). Discrete foci of virus-infected cells are indicated with blue arrows in rhesus macaques with more widespread distribution of ZIKV RNA in tamarins, particularly tear ducts and salivary gland LN, (circled).

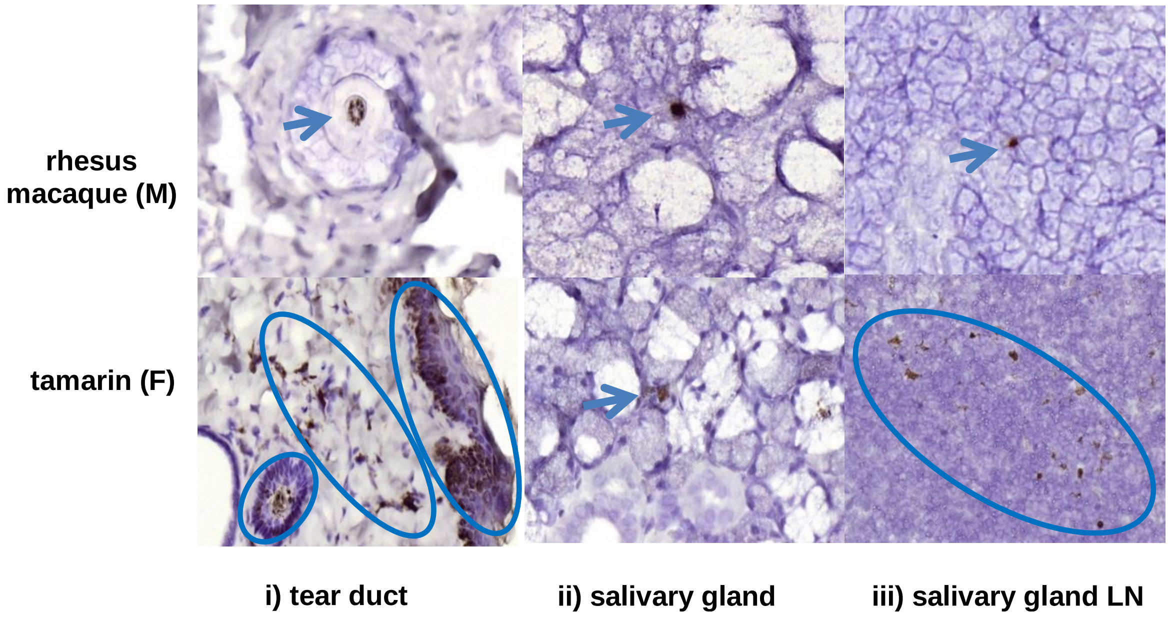

Figure S4. Immunohistochemical staining for NS1 protein in peripheral nerves (lower limbs) All images x40 magnification shown for section across areas of lower limb in rhesus macaques (left panels) and tamarins (right panels) at 3, 42 and 100 days post-infection (dpi). Clusters of nerve fibre cells are visible at 3dpi.

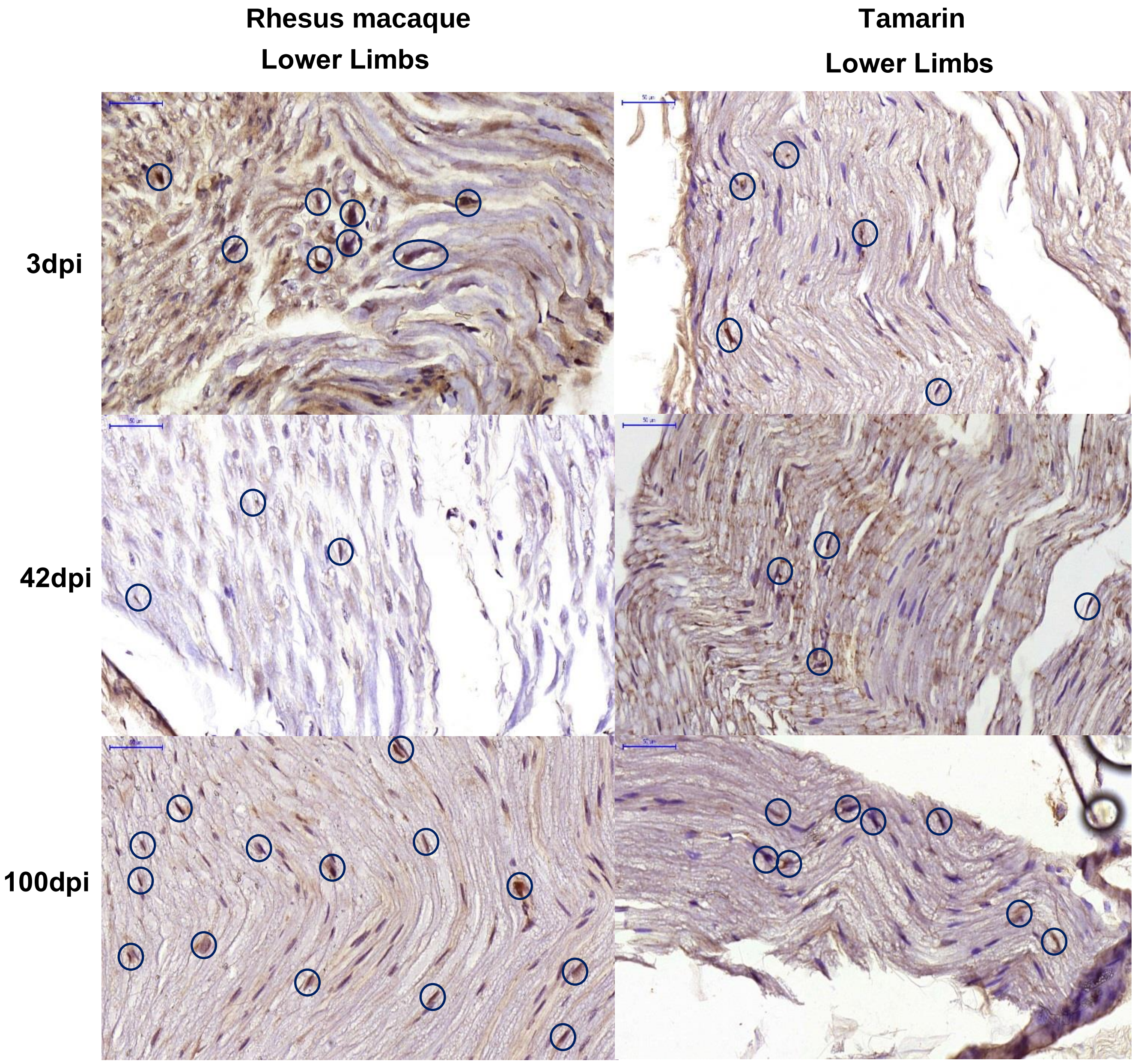

Figure S5. Distribution of DC-SIGN. DC-SIGN staining by immunohistochemistry in rhesus macaques and red-bellied tamarins at 3, 42 and 101 days post-infection (dpi) at the inoculation site, shoulder and ankle compared to control tissue sections.

a

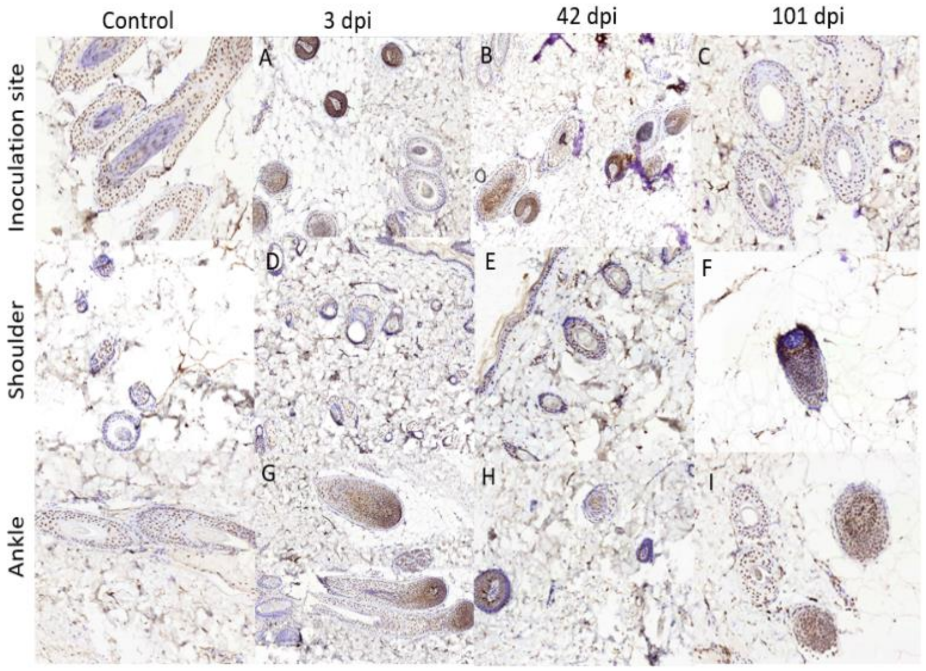

Rhesus macaque

b

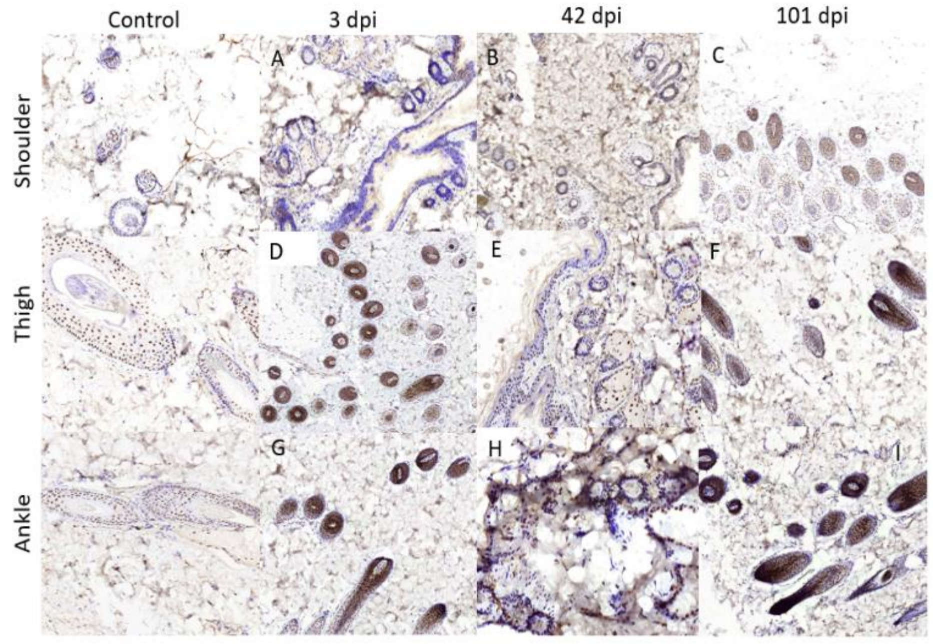

Tamarin

Figure S6. Distribution of CD68+ macrophages. Macrophage marker CD68 staining by immunohistochemistry in Rhesus macaques and red-bellied tamarins at 3, 42 and 101 days post-infection (dpi) at the inoculation site, shoulder and ankle compared to similarly processed control tissue sections.

a

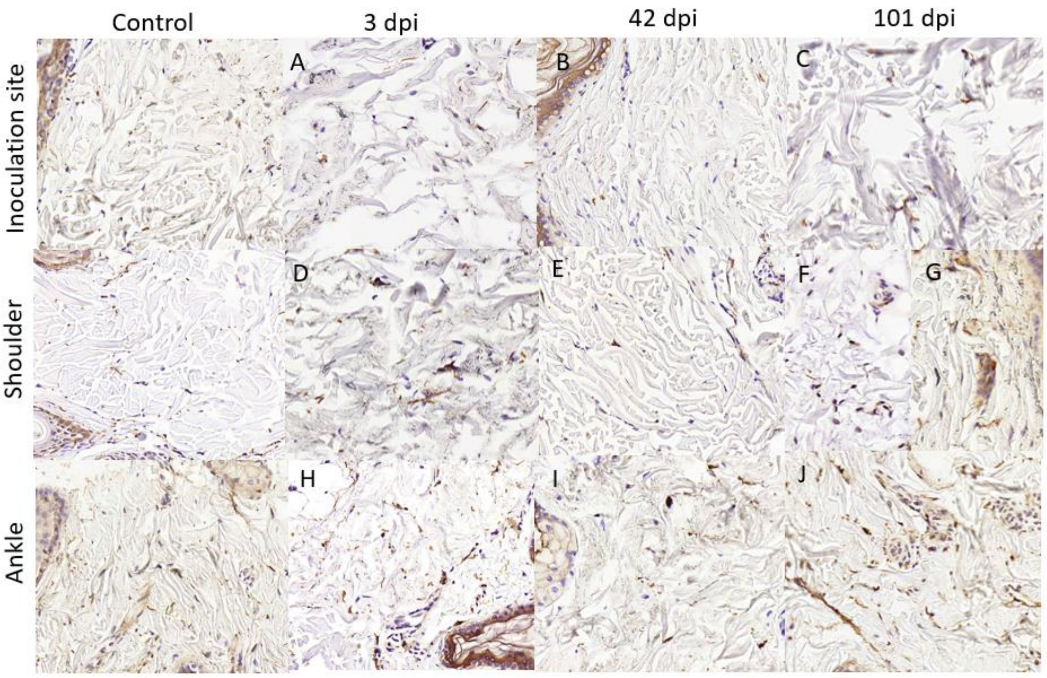

Rhesus macaque

b

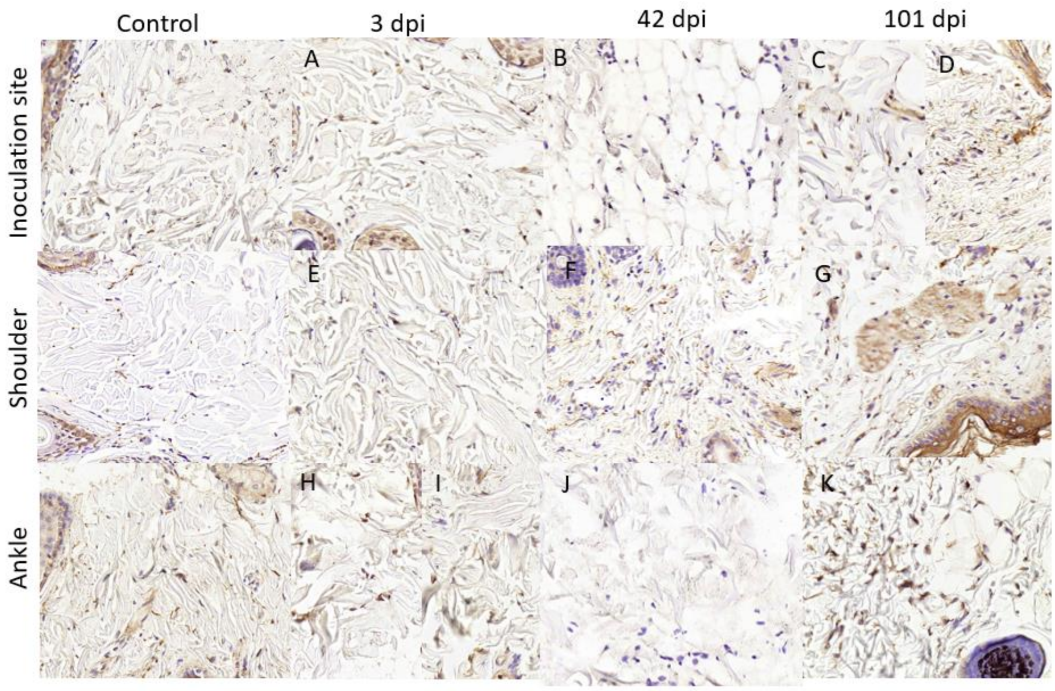

Tamarin
